# The Anatomy of the Central Nervous System of Man, and of Vertebrates in General

**Published:** 1900-09

**Authors:** 


### Reviews of Books.

**The Anatomy of the Central Nervous System of Man, and of Vertebrates in General.** By Prof. LUDWIG EDINGER, M.D. Translated from the Fifth German Edition by WINFIELD S. HALL, M.D. Pp. xi., 446. Philadelphia: The F. A. Davis Company. 1899.

This is an old friend in an enlarged and revised form. Its unquestionable value as an introduction to the minute study of the nervous system entitles it to the consideration of everyone interested in that intricate mechanism.

Its greatest merit lies in the fact that it teaches us the anatomy of the brain and its addenda from the point of view of comparative anatomy, which is certainly the only true method. The structure of the nervous system is placed before us in its simplest condition, and step by step elaborated in the animal series till its complexity reaches its climax in man. This well-nigh unintelligible maze, as usually approached in text-books, has now been for the most part rendered perfectly clear by the method of Edinger, and he has brought to bear on it a mass of knowledge which perhaps he alone would be able to supply.

How comparatively simple is the structure of the spinal cord and that of the medulla oblongata when dealt with as it is here; and how forcibly one is struck with the fact that after all the medulla is but a specialised part of a column of which the spinal cord forms the greatest part! It is shown that upon a collection of nerve cells and fibres, intrinsic to what we call the cord, is built a superstructure which places that simple cord in relation with parts in front of it, adds to its bulk, complexity, and utility, and which at the same time reduces its almost regal position in some lower vertebrates to that of comparative serfdom as we find it in man! Instead of being confronted with a great complex of tracts, commissures, and cells, as is most commonly the case, we are introduced step by step to cell cohesions, to tracts formed by posterior nerve-roots, to intrinsic tracts—short commissural, to actual true commissures, and finally to three or four great tracts which place the cord in relation to that part which we usually term the higher brain. Thus we are introduced to such important pathways as the *tractus cerebello-spinalis*, which places the cord in relation with the cerebellum in all animals from cartilaginous and bony fishes to mammals; the *fasciculus longitudinalis posterior*, which connects the thalamencephalon and the various motor nuclei with the grey matter of the cord. Then that important sensory tract, the *fillet*, is introduced to us, perhaps not so happily as the others, as from the description one would get the impression that it was mainly a downwardly conducting path; and, moreover, from what follows it would seem that the tract of Gowers would seem to be included in this system, although such an impression is excluded when that tract is itself considered. Finally, we are brought face to face with that characteristically mammalian tract, the *pyramidal tract*. Such an impression as the above tempts one to read further, and we find the higher parts treated in the same rational fashion. We confess we should like to have seen further reference to well-known work on the morphology of the cerebral convolutions, especially that of our fellow-countryman Cunningham, and of our kinsman Elliot-Smith, whose admirable work on the lower mammalian brain has deservedly excited the greatest admiration, and we should have liked to have had the work rendered into more elegant English than it has been. Certainly it is no easy task to combine free and literal translation of the German language, but we do think that something better than "fundament" might have been made of the word *anlage*. To be faced with "*Kupffer's fundament*" and with "*Froriep's fundament*" is unpleasant, to say the least; and we doubt if Edinger really meant to inform us that "The fibres thus passing through the corpus striatum lie in two masses. . . . The outer one is called the *nucleus lentiformis* and the inner one the *nucleus caudatus*." We think, too, that many of the illustrations, especially those concerning the comparative and embryological side, might be much improved. In many cases the descriptive letterpress is printed on the figures themselves, and the letters are so small and so confused with the figure that it is next to impossible to decipher them. On the whole, however, the illustrations are excellent, despite these drawbacks, which bear no comparison with the great merit of the work. This work stands alone in giving a really intelligible, concise view of the structure of the nervous system: as such it will continue to maintain the confidence which has hitherto been placed in it in its more modest form; in its enlarged and revised state, we shall be much mistaken if that confidence be not much enhanced. No practitioner and certainly no teacher ought to be without it.

